# Fingolimod Phosphate Attenuates Oligomeric Amyloid β–Induced Neurotoxicity via Increased Brain-Derived Neurotrophic Factor Expression in Neurons

**DOI:** 10.1371/journal.pone.0061988

**Published:** 2013-04-12

**Authors:** Yukiko Doi, Hideyuki Takeuchi, Hiroshi Horiuchi, Taketo Hanyu, Jun Kawanokuchi, Shijie Jin, Bijay Parajuli, Yoshifumi Sonobe, Tetsuya Mizuno, Akio Suzumura

**Affiliations:** Department of Neuroimmunology, Research Institute of Environmental Medicine, Nagoya University, Furo-cho, Chikusa-ku, Nagoya, Japan; University of Florida, United States of America

## Abstract

The neurodegenerative processes that underlie Alzheimer's disease are mediated, in part, by soluble oligomeric amyloid β, a neurotoxic protein that inhibits hippocampal long-term potentiation, disrupts synaptic plasticity, and induces the production of reactive oxygen species. Here we show that the sphingosine-1-phosphate (S1P) receptor (S1PR) agonist fingolimod phosphate (FTY720-P)-a new oral drug for multiple sclerosis-protects neurons against oligomeric amyloid β-induced neurotoxicity. We confirmed that primary mouse cortical neurons express all of the S1P receptor subtypes and FTY720-P directly affects the neurons. Treatment with FTY720-P enhanced the expression of brain-derived neurotrophic factor (BDNF) in neurons. Moreover, blocking BDNF-TrkB signaling with a BDNF scavenger, TrkB inhibitor, or ERK1/2 inhibitor almost completely ablated these neuroprotective effects. These results suggested that the neuroprotective effects of FTY720-P are mediated by upregulated neuronal BDNF levels. Therefore, FTY720-P may be a promising therapeutic agent for neurodegenerative diseases, such as Alzheimer's disease.

## Introduction

Alzheimer's disease (AD) is the most common cause of dementia [1], [2]. Senile plaques consisting of insoluble fibrillar amyloid β (Aβ) are pathologic hallmarks of AD. Aβ is formed after sequential cleavage of amyloid precursor protein and is secreted to the extracellular space. Aβ has a strong fibrillogenic property, and soluble Aβ monomers gradually convert to oligomers and ultimately to insoluble fibrils. Soluble oligomeric Aβ (oAβ) is considered to be more important in the pathogenesis of AD than fibrillar Aβ, because oAβ is more neurotoxic [3], [4]. Naturally secreted oAβ inhibits hippocampal long-term potentiation and disrupts of synaptic plasticity [2]. In addition, oAβ induces elevation of reactive oxygen species levels in neurons, leading to neuronal death [5].

Fingolimod is a new oral drug for multiple sclerosis [6]–[9]. Fingolimod was synthesized by modifying myriocin, which is derived from *Isaria sinclairii* [10]. Because fingolimod is a structural analogue of sphingosine, it is phosphorylated by sphingosine kinase *in vivo*. Once phosphorylated, fingolimod binds to sphingosine-1-phosphate (S1P) receptor 1 (S1P1) on the surface of lymphocytes, and the receptors are internalized. These lymphocytes can no longer move out of lymphoid tissues. Therefore, fingolimod phosphate (FTY720-P) prevents autoreactive lymphocytes from infiltrating the central nervous system (CNS) and suppresses subsequent neuroinflammation [11]–[13].

Many studies have shown that S1PRs are widely expressed in many cell types, including neurons, astrocytes, microglia, and oligodendrocytes [14]. The functions of these receptors have not been elucidated completely, however. There are five S1PR subtypes: S1P_1_, S1P_2_, S1P_3_, S1P_4_, and S1P_5_. FTY720-P binds to all S1PR subtypes except S1P_2_ [15], [16]. Previous studies demonstrated that FTY720-P directly induced oligodendrocytes to promote remyelination [17] and enhanced neuroprotective effects in astrocytes [18]. Moreover, we have recently showed that FTY720-P augmented microglial neuroprotective effects by downregulation of pro-inflammatory cytokines and upregulation of neurotrophic factors [19]. However, it is still uncertain whether FTY720-P directly affects neurons. S1P reportedly promotes neurogenesis from proliferation of neuronal progenitor cells [20]. S1P also contributes to the migration of neuronal stem/progenitor cells [21]. Therefore, FTY720-P may directly affect neurons. Here, by assessing the effects of FTY720-P on neurons, we show that FTY720-P directly upregulates neuronal brain-derived neurotrophic factor (BDNF) production to attenuate oAβ-induced neurotoxicity.

## Methods

### Preparation of fingolimod phosphate

Fingolimod phosphate (FTY720-P) was kindly provided by Tanabe Mitsubishi Pharma (Osaka, Japan). FTY720-P was dissolved in 80% ethanol containing 2 mM NaOH.

### Preparation of oligomeric Aβ1-42

Soluble oligomeric Aβ1-42 (oAβ1-42) was prepared as described previously [4], [22]. Briefly, synthetic human Aβ1-42 (Peptide Institute, Osaka, Japan) was dissolved in 100% 1,1,1,3,3,3-hexafluoro-2-propanol and dried to completely remove the solvent. The obtained film was resuspended in dimethyl sulfoxide, and diluted with Dulbecco's Modified Eagle Medium/F12 (Invitrogen, Carlsbad, CA, USA) at a concentration of 100 µM. This solution was incubated at 4°C for 24 h to obtain oAβ. A final concentration of 5 µM oAβ1-42 was used for all experiments. The oligomerization of oAβ was characterized by Western blotting as below.

### Neuronal cultures

Protocols for animal experiments were approved by the Animal Experiment Committee of Nagoya University (permission number: 12122). Primary mouse cortical neurons were prepared as described previously [4], [23]. Briefly, cerebral cortices were isolated from embryonic day 17 C57BL/6J mouse embryos, minced and treated with dissociation solution (Sumitomo Bakelite, Akita, Japan). Resulting neurons were resuspended in Nerve Culture Medium™ (Sumitomo Bakelite), plated on polyethylenimine-coated glass coverslips (Asahi Techno Glass, Chiba, Japan) at a density of 5×103 cells/well in 96-well multidishes, 5×104 cells/well in 24-well multidishes or 5×105 cells/well in 6-well multidishes, and incubated at 37°C in an atmosphere containing 5% CO2 at 100% humidity. The purity of the cultures was>95% based on NeuN-specific immunostaining. Neurons were used at 14 days *in vitro* for the following assessments.

### RNA extraction and reverse transcription-polymerase chain reactions (RT-PCRs)

To assess the neuronal expression of S1PRs, neurons at 14 days *in vitro* were stimulated with 5 µM oAβ1-42 for 6 h. Expression of mRNA encoding S1P_1_, S1P_2_, S1P_3_, S1P_4_, and S1P_5_ was detected using RT-PCRs. Total RNA was isolated with an RNeasy Mini Kit (Qiagen, Valencia, CA, USA) and reverse transcribed with SuperScript II (Invitrogen). PCRs were performed using Blend Taq (Toyobo, Osaka, Japan). The following sense and antisense primers were used:

S1P_1_ sense: 5′-GACCATGGCATTAAACTGACTT-3′

S1P_1_ antisense: 5′-TGTAGTTTCATCTTCAGCATGG-3′

S1P_2_ sense: 5′-AAGTTCCACTCAGCAATGTACC-3′

S1P_2_ antisense: 5′-TAGATGACAGGATTGAGCAGTG-3′

S1P_3_ sense: 5′-TCTTCTTGGTCACCTGTAGCTT-3′

S1P_3_ antisense: 5′-TCGATGAGGAAGAGGATAAAAA-3

S1P_4_ sense: 5′-GTGTACTACTGCCTGCTGAAC-3′

S1P_4_ antisense: 5′-GATGAGAGGATTAATGGCTGAG-3

S1P_5_ sense: 5′-GCTTTGGTTTGCGCGTGAG-3′

S1P_5_ antisense: 5′-GGCGTCCTAAGCAGTTCCAG-3

GAPDH sense: 5′-TGTGTCCGTCGTGGATCTGA-3′

GAPDH antisense: 5′-CCTGCTTCACCACCTTCTTGA-3′

To assess the neuronal expression of neurotrophic factors, neurons at 14 days *in vitro* were stimulated with 1–100 pM FTY720-P for 6 h. Total RNA was isolated with an RNeasy Mini Kit (Qiagen) and reverse transcribed with SuperScript II (Invitrogen). Expression levels of mRNA encoding BDNF, nerve growth factor (NGF), and neurotrophin-3 (NT-3) were evaluated using quantitative PCR (qPCR), which was performed on the cDNA using a Rotor-Gene Q (Qiagen) and a TaqMan® Gene Expression Master Mix (Applied Biosystems, Foster City, CA, USA). The mouse gene-specific primers and probes were obtained from Applied Biosystems: BDNF (Mm04230607_s1), NGF (Mm00443039_m1), NT-3 (Mm00435413_s1), HPRT1 (Mm01545399_m1) and GAPDH (Mm99999915_g1). Gene expression values were determined by using the ΔΔC_T_ method. The genes of interest were standardized to the geometric mean of HPRT1 and GAPDH. Assays were carried out in six independent trials.

### Western blotting

To confirm the oligomerization, oAβ1-42 was also dissolved in Laemmli sample buffer. Cell lysates were used to examine S1PR subtypes and BDNF production. The neurons at 14 days *in vitro* were treated with 5 µM oAβ or 100 pM FTY720-P for 24 h. Cells were lysed in lysis buffer (50 mM Tris-HCl at pH 7.6, 1% Nonidet P-40, 150 mM NaCl, 2 mM EDTA, and protease inhibitor mixture (Complete Mini EDTA-free, Roche, Germany)). Soluble fractions were collected following centrifugation for 5 min at 10,000 rpm and the protein concentration was determined in a bicinchoninic acid assay (Thermo Fisher Scientific, Rockford, IL, USA). The soluble fractions of the cell lysates or neuronal culture supernatant were dissolved in Laemmli sample buffer.

Forty micrograms of cell lysate protein or 300 ng of oAβ1-42 dissolved in Laemmli sample buffer were separated on 4–20% SDS-polyacrylamide gels (Mini-Protean TGX™, Bio-Rad, Hercules, CA, USA), and transferred to Hybond-P polyvinylidene difluoride membranes (GE Healthcare, Buckingham, UK). The membranes were blocked with 1% skim milk in Tris-buffered saline containing 0.05% Tween-20 for 1 h at room temperature, and then incubated overnight at 4°C with rabbit anti-S1P1 polyclonal antibodies (1∶200; Cayman Chemical, Ann Arbor, MI, USA), rabbit anti-S1P2 polyclonal antibodies (1∶500; Cayman Chemical), rabbit anti-S1P3 polyclonal antibodies (1∶200; Cayman Chemical), rabbit anti-S1P4 polyclonal antibodies (1∶100; Cayman Chemical), rabbit anti-S1P5 polyclonal antibodies (1∶200; Cayman Chemical), rabbit anti-BDNF polyclonal antibodies (N-20) (1∶200; Santa Cruz Biotechnology, Santa Cruz, CA, USA), mouse anti-Aβ monoclonal antibodies (6E10) (1∶1000; Covance, Princeton, NJ, USA), mouse anti-GAPDH monoclonal antibodies (3H12) (1∶1000; MBL, Nagoya, Japan), or mouse anti-β-actin monoclonal antibodies (AC-15) (1∶2000; Sigma) followed by horseradish peroxidase–conjugated secondary antibodies (1∶5000; GE Healthcare, Buckingham, UK) for 1 h at room temperature. The signals were visualized using SuperSignal West Pico chemiluminescent substrate (Thermo Fisher Scientific), and quantified using a CS Analyzer 3.0 system (Atto, Tokyo, Japan). Assays were carried out in six independent trials.

### Enzyme-linked immunosorbent assay (ELISA)

BDNF was measured using an ELISA kit according to the manufacture's protocol (Emax ImmunoAssay Systems; Promega, Madison, WI, USA). Neurons at 14 days *in vitro* were treated with 5 µM oAβ or 100 pM FTY720-P for 24 h. Cell lysates were obtained with lysis buffer and soluble fractions were collected following centrifugation for 5 min at 10,000×*g*. Assays were carried out in three independent trials.

### Drug treatment

Neurons at 14 days *in vitro* were treated with 1–100 pM FTY720-P or 250–1000 pg/ml mouse recombinant BDNF (Promega) from 3 h before oAβ1-42 stimulation. To block BDNF-TrkB signaling, 0.01–1 µg/ml BDNF scavenger (TrkB Fc chimera, R&D systems, Minneapolis, MN, USA) or 0.001–10 µg/ml TrkB inhibitor (ANA-12; Sigma, St. Louis, MO, USA), and 1 µM ERK1/2 inhibitor (U0126; Millipore, Billerica, MA, USA) was added from 1 h before FTY720-P treatment. After stimulation with 5 µM oAβ1-42 for 24 h, neuronal survival was assessed as below.

### Immunocytochemistry

Neurons were fixed with 4% paraformaldehyde for 10 min, permeabilized with 0.1% Triton X-100, and blocked with 5% normal goat serum in phosphate-buffered saline for 1 h at room temperature. Neurons were stained with rabbit anti-S1P1 polyclonal antibodies (1∶100; Cayman Chemical), rabbit anti-S1P2 polyclonal antibodies (1∶100; Cayman Chemical), rabbit anti-S1P3 polyclonal antibodies (1∶100; Cayman Chemical), rabbit anti-S1P4 polyclonal antibodies (1∶100; Cayman Chemical), rabbit anti-S1P5 polyclonal antibodies (1∶100; Cayman Chemical), rabbit anti-microtubule-associated protein 2 (MAP-2) polyclonal antibodies (1∶1000; Millipore, Billerica, MA, USA) and Aβ was stained with mouse anti-Aβ monoclonal antibody (4G8) (1∶1000; Chemicon, Billerica, MA, USA). The samples were observed using a deconvolution fluorescence microscope system (BZ-8000; Keyence, Osaka, Japan).

### Assessments of neuronal survival

Neuronal survival was assessed by the number of MAP-2-positive neurons and 2-(2-methoxy-4-nitrophenyl)-3-(4-nitrophenyl)-5-(2,4-disulfophenyl)-2H-tetrazolium (WST-8) assay. To count MAP-2-positive neurons and normalized based on results observed with untreated neurons as described previously [24]. Viable neurons stained strongly with an anti-MAP-2 antibody, whereas damaged neurons showed much weaker staining. The number of MAP-2-positive neurons was counted in 10 random fields per well. More than 200 cells were examined in three independent trials. The number of untreated viable neurons was normalized to 100%.

WST-8 assay was carried out in six independent trials using Cell Counting Kit-8 (Dojindo, Kumamoto, Japan) according to the manufacture's instruction. Absorbance at 450 nm was measured in a multiple plate reader.

### Statistical analysis

Statistical significance was analyzed with Student's *t*-test or one-way analysis of variance followed by post-hoc Tukey's test using GraphPad Prism version 5.0 (GraphPad Software, La Jolla, CA, USA).

## Results

### Neurons express S1PRs

**Figure 1:**
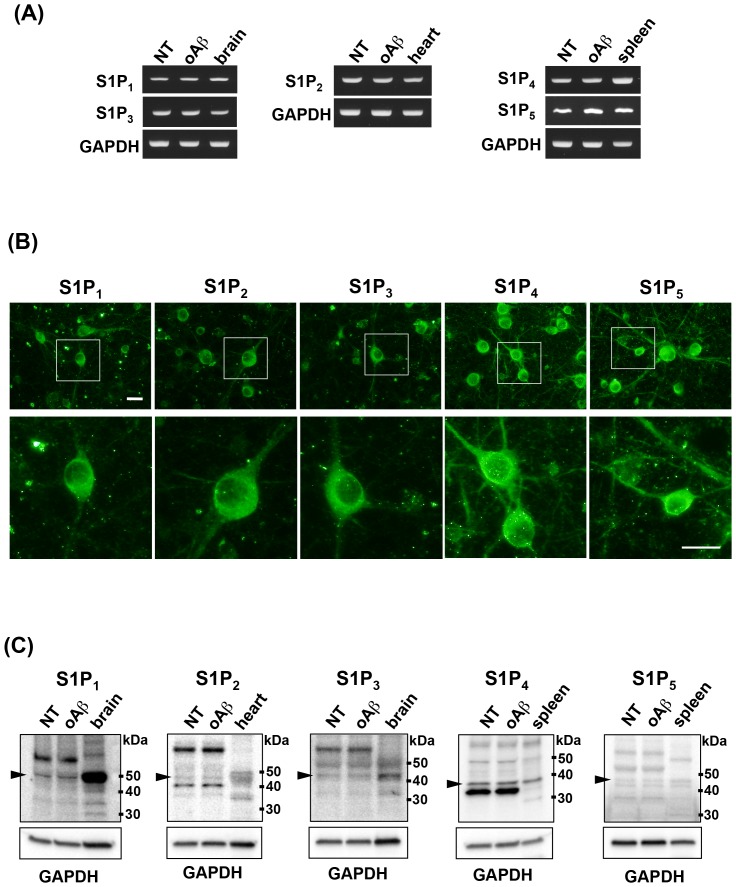
Neurons express all S1PR subtypes. (A) Representative RT-PCR data for S1PRs. Neurons constitutively expressed all S1PR subtypes regardless of oAβ–stimulation. (B) Immunostaining data for S1PRs in untreated neurons. Neurons constitutively expressed all S1PR subtypes. Scale bar, 20 µm. (C) Western blotting data for S1PRs. Arrowheads indicate the bands for S1PRs. Neurons constitutively expressed all S1PR subtypes regardless of oAβ–stimulation. NT, no treatment; oA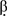 5 µM oAβ1-42 treatment. The positive control samples used for each S1PR were as follows; brain for S1P_1_ and S1P_3_; heart for S1P_2_; spleen for S1P_4_ and S1P_5_.

First, we examined S1PR expression on primary cortical neurons. RT-PCR analysis revealed that cortical neurons constitutively expressed all S1PR subtypes regardless of stimulation with 5 µM oAβ1-42 (Figure 1A). Immunostaining for S1PRs depicted that cortical neurons expressed all S1PR subtypes (Figure 1B). Western blotting analysis also showed that cortical neurons constitutively expressed all S1PR subtypes (Figure 1C, arrowheads), which is consistent with the RT-PCR data, although multiple non-specific bands were also detected. Treatment with 5 µM oAβ1-42 did not alter the expression levels of S1PRs.

### FTY720-P suppresses oAβ1-42-induced neurotoxicity

**Figure 2:**
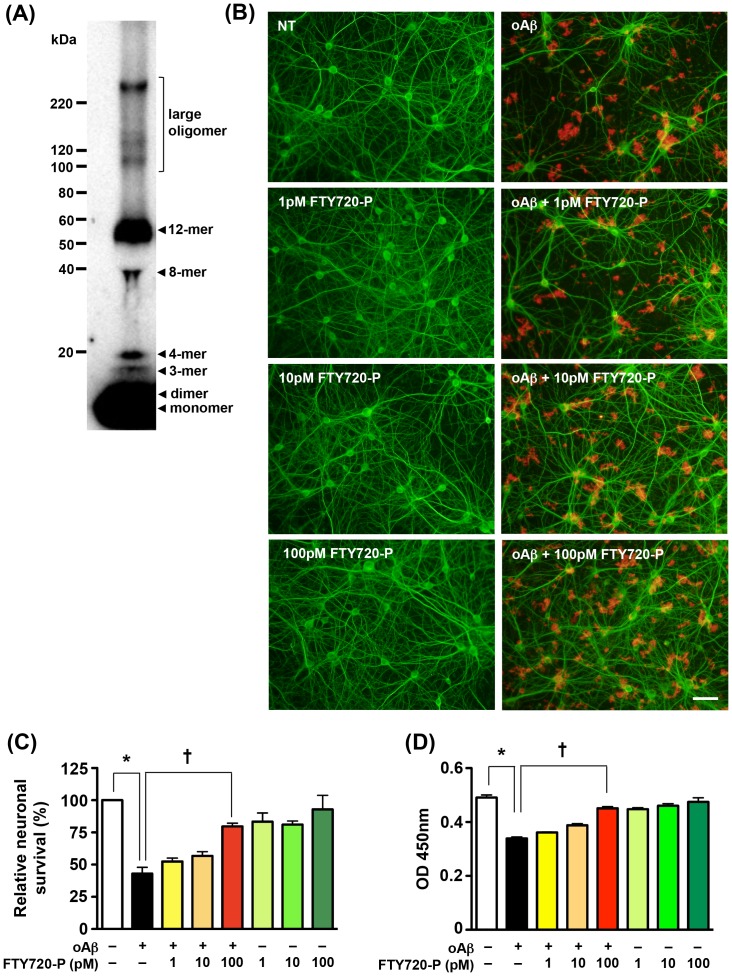
FTY720-P suppressed oAβ–induced neurotoxicity. (A) Western blotting image for characterization of oAβ ˜ Data showed Aβ1-42 oligomerization composed with small (monomer, dimer, 3-mer, 4-mer, 8-mer, and 12-mer) and large oligomers. (B) Fluorescent microscopic images of mouse primary cortical neuron cultures. Treatment with FTY720-P was neuroprotective against oAβ–mediated toxicity. Neurons were stained with anti–MAP-2 (green) antibodies and Aβ was stained with 4G8 antibodies` (red). Scale bar: 50 µm. NT, no treatment; oA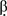 5 µM oAβ1-42treatment. (C) Relative neuronal survival. The number of viable neurons (MAP-2–positive neurons) was quantified relative to results observed with untreated neurons. FTY720-P rescued neurons against oAβ–mediated toxicity. oA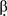 5 µM oAβ1-42treatment. *, *P*<0.001; †, *P*<0.001. Values are means±SEM (n = 3). (D) WST-8 assay. FTY720-P enhanced neuronal survival against oAβ–mediated toxicity. oA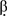 5 µM oAβ1-42treatment. *, *P*<0.001; †, *P*<0.001. Values are means ± SEM (n = 6).

We confirmed the oligomerization of oAβ1-42 (Figure 2A). As we had shown previously [4], [24], Western blotting data demonstrated that oAβ1-42 we used mainly consisted oligomers, but not fibrillar Aβ. We then examined the effects of FTY720-P on oAβ1-42 induced neurotoxicity. Treatment with 1–100 pM FTY720-P alone did not affect neuronal survival (Figure 2B, 1 pM FTY720-P, 10 pM FTY720-P, and 100 pM FTY720-P; Figure 2C and 2D, green columns). Next, neuronal cultures were treated with 1–100 pM FTY720-P for 3 h prior to the addition of 5 µM oAβ1-42 for 24 h. Treatment with 5 µM oAβ1-42 for 24 h resulted in severe neurotoxicity (Figure 2B, oAβ; Figure 2C and 2D, black columns), which agrees with previous reports [3], [4], [25]. However, treatment with 100 pM FTY720-P protected the neurons against oAβ–induced toxicity (Figure 2B, oAβ+100 pM FTY720-P; Figure 2C and 2D, red columns).

### FTY720-P induces BDNF production

**Figure 3:**
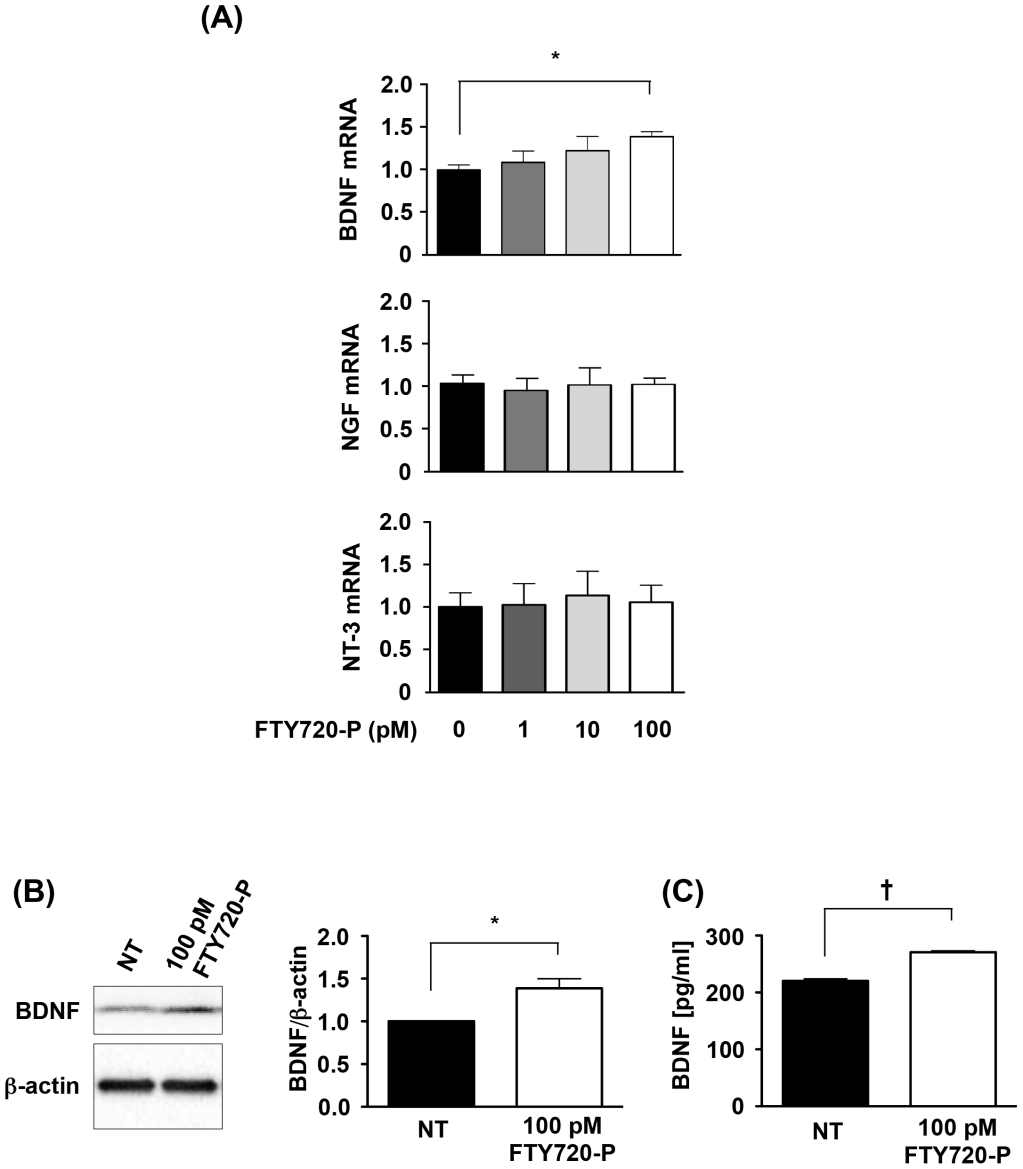
FTY720-P enhanced BDNF production. (A) qRT-PCR data for neurotrophic factors in neurons. FTY720-P significantly upregulated mRNA expression levels of BDNF. *, *P*<0.05. Values are means ± SEM (n = 6). (B) Western blotting data for BDNF production. FTY720-P significantly enhanced neuronal BDNF production. BDNF levels are quantified relative to those in untreated neurons. NT, no treatment. *, *P*<0.05. Values are means ± SEM (n = 6). (C) ELISA data for BDNF production. BDNF levels significantly increased in response to FTY720-P. NT, no treatment. †, *P*<0.001. Values are means ± SEM (n = 3).

Next, we examined whether FTY720-P exerts neuroprotective effects by upregulating neurotrophic factors like microglia [19]. We assessed mRNA expression levels of neuronal BDNF, NGF, and NT-3 using qPCR. Interestingly, FTY720-P increased mRNA expression levels of BDNF, whereas it did not significantly affect those of NGF and NT-3 (Figure 3A). Neuronal BDNF production was also significantly enhanced by 100 pM FTY720-P based on results obtained with Western blotting and ELISAs (Figure 3B, C).

### Neuroprotective effects of FTY720-P depend on BDNF-TrkB signaling

BDNF exerts neuroprotective effects through its receptor TrkB [26]. To examine whether FTY720-P is protective via neuronal BDNF expression, we blocked BDNF-TrkB signaling using a TrkB–Fc chimera as a BDNF scavenger and ANA-12 as a TrkB inhibitor.

**Figure 4:**
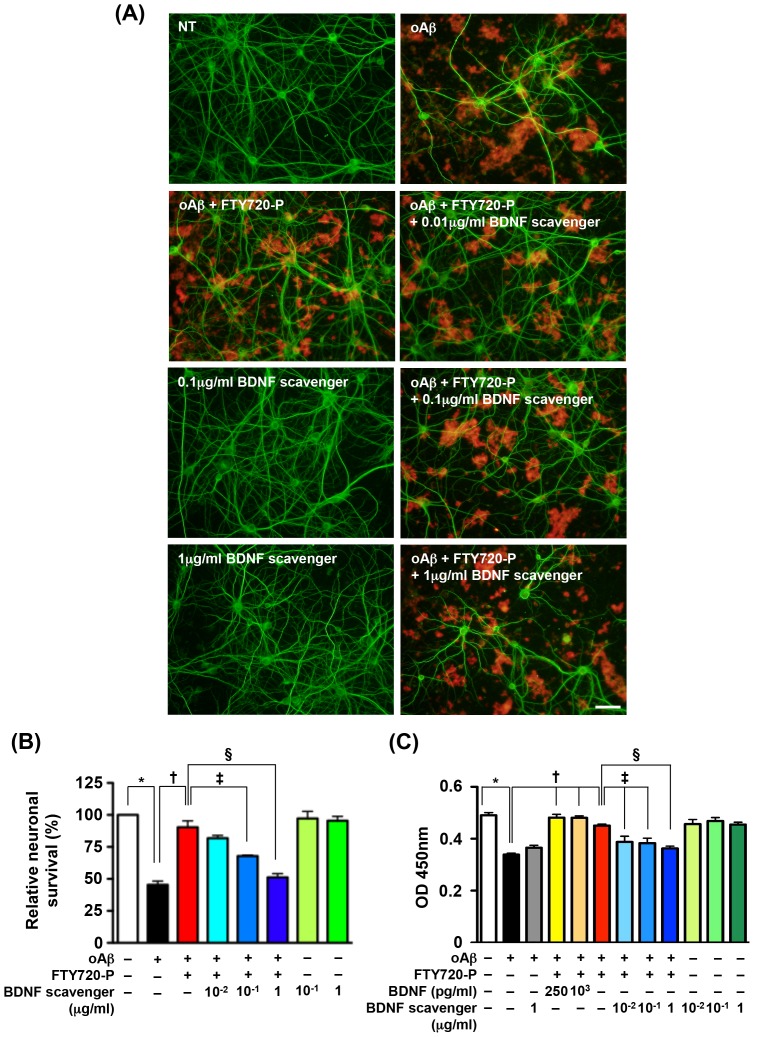
Neuroprotective effects of FTY720-P depended on BDNF. (A) Fluorescent microscopic images of mouse primary cortical neuron cultures. The BDNF scavenger canceled the neuroprotective effects of FTY720-P. Neurons were stained with anti–MAP-2 antibodies (green) and Aβ was stained with 4G8 antibodies (red). Scale bar: 50 µm. NT, no treatment; oA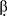 5 µM oAβ1-42treatment; FTY720-P, 100 pM FTY720-P treatment. (B) Relative neuronal survival. The number of viable neurons (MAP-2–positive neurons) was quantified relative to results obtained with untreated neurons. The BDNF scavenger dose-dependently suppressed the neuroprotective effects of FTY720-P. oA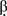 5 µM oAβ1-42treatment; FTY720-P, 100 pM FTY720-P treatment. *, *P*<0.001; †, *P*<0.001; ‡, *P*<0.01; §, *P*<0.001. Values are means ± SEM (n = 3). (C) WST-8 assay. Addition of BDNF enhanced neuronal survival against oAβ–mediated toxicity to the same extent of FTY720-P. The BDNF scavenger reversed the neuroprotective effects of FTY720-P in a dose-dependent manner, whereas it did not enhance oAβ–mediated neurotoxicity. oA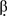 5 µM oAβ1-42treatment; FTY720-P, 100 pM FTY720-P treatment. *, *P*<0.001; †, *P*<0.001; ‡, *P*<0.01; §, *P*<0.001. Values are means ± SEM (n = 6).

We confirmed that treatment with BDNF scavenger alone did not alter neuronal survival (Figure 4A, 0.1 µg/ml BDNF scavenger and 1 µg/ml BDNF scavenger; Figure 4B and 4C, green columns). The BDNF scavenger dose-dependently suppressed the neuroprotective effects of FTY720-P (Figure 4A, oAβ+FTY720-P+0.01 µg/ml BDNF scavenger, oAβ+FTY720-P+0.1 µg/ml BDNF scavenger and oAβ+FTY720-P+1 µg/ml BDNF scavenger; Figure 4B and 4C, blue columns). BDNF scavenger per se did not enhance oAβ–mediated neurotoxicity (Figure 4C, gray column). Morevover, addition of BDNF prevented oAβ–mediated neurotoxicity to the same extent of FTY720-P (Figure 4C, yellow and orange columns).

**Figure 5:**
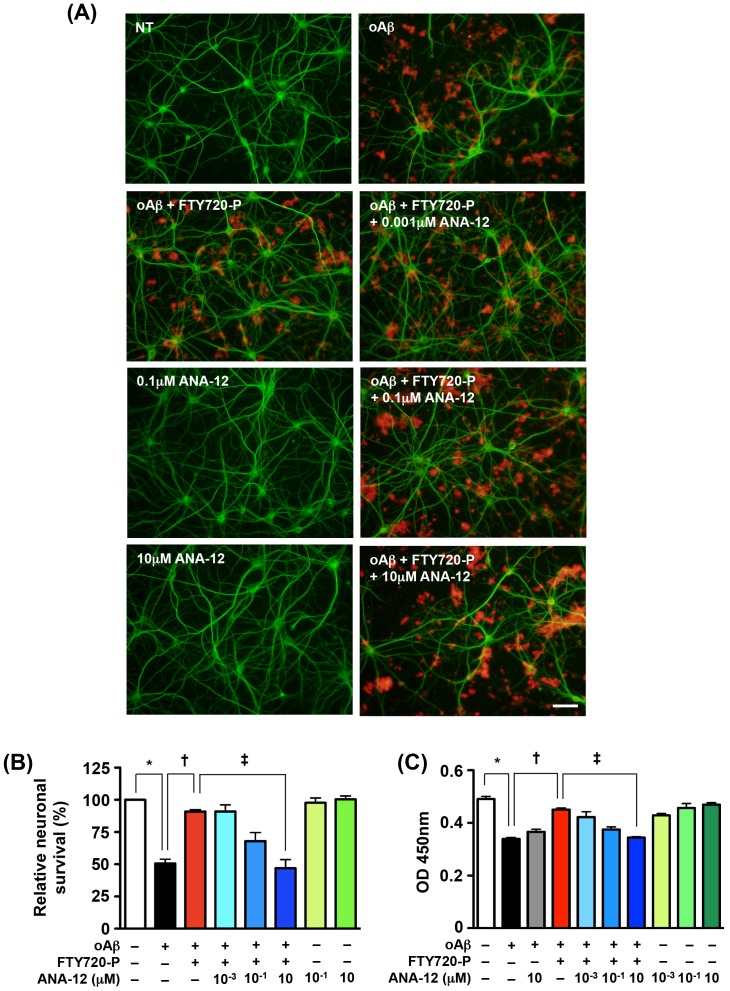
Activation of TrkB by BDNF is critical for the neuroprotective effects of FTY720-P. (A) Fluorescent microscopic images of mouse primary cortical neuron cultures. The TrkB inhibitor ANA-12 ablated the neuroprotective effects of FTY720-P against oAβ-induced neurotoxicity. Neurons were stained with anti–MAP-2 antibodies (green) and Aβ was stained with 4G8 antibodies (red). Scale bar: 50 µm. NT, no treatment; oA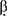 5 µM oAβ1-42treatment; FTY720-P, 100 pM FTY720-P treatment. (B) Relative neuronal survival. The number of viable neurons (MAP-2–positive neurons) was quantified relative to results observed in untreated neurons. TrkB inhibition almost completely inhibited the neuroprotective effects of FTY720-P. oA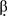 5 µM oAβ1-42treatment; FTY720-P, 100 pM FTY720-P treatment. *, *P*<0.001; †, *P*<0.001; ‡, *P*<0.001. Values are means ± SEM (n = 4). (C) WST-8 assay. Blocking TrkB almost completely reversed the neuroprotective effects of FTY720-P, whereas it did not enhance oAβ–mediated neurotoxicity. oA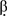 5 µM oAβ1-42treatment; FTY720-P, 100 pM FTY720-P treatment. *, *P*<0.001; †, *P*<0.001; ‡, *P*<0.001. Values are means ± SEM (n = 6).

We confirmed that treatment with the TrkB inhibitor ANA-12 alone did not alter neuronal survival (Figure 4A, 0.1 µM ANA-12 and 10 µM ANA-12; Figure 4B and 4C, green columns). 10 µM ANA-12 almost completely ablated the neuroprotective effects of FTY720-P (Figure 5A, oAβ+FTY720-P+10 µM ANA-12; Figure 5B and 5C, blue columns). ANA-12 per se did not enhance oAβ–mediated neurotoxicity (Figure 5C, gray column). These results demonstrated that the neuroprotective effects of FTY720-P depend on BDNF-TrkB signaling.

### The ERK1/2 pathway contributes to FTY720-P–mediated neuroprotection

**Figure 6:**
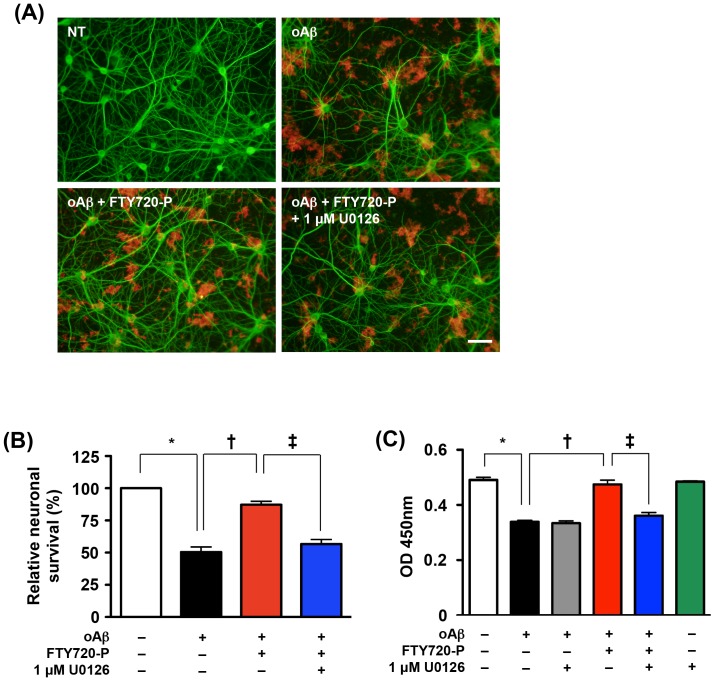
The neuroprotective effect of FTY720-P require ERK1/2 signaling. (A) Fluorescent microscopic images of mouse primary cortical neuron cultures. ERK1/2 inhibitor U0126 almost completely suppressed FTY720-P–mediated protection against oAβ-induced neurotoxicity. Neurons were stained with anti–MAP-2 antibodies (green) and Aβ was stained with 4G8 antibodies (red). Scale bar: 50 µm. (B) Relative neuronal survival. The number of viable neurons (MAP-2–positive neurons) was quantified relative to results observed in untreated neurons. ERK1/2 inhibition almost completely ablated the neuroprotective effects of FTY720-P. oA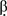 5 µM oAβ1-42treatment; FTY720-P, 100 pM FTY720-P treatment. *, *P*<0.001; †, *P*<0.001; ‡, *P*<0.001. Values are means ± SEM (n = 3). (C) WST-8 assay. Blocking ERK1/2 almost completely canceled the neuroprotective effects of FTY720-P, whereas it did not enhance oAβ–mediated neurotoxicity. oA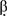 5 µM oAβ1-42 treatment; FTY720-P, 100 pM FTY720-P treatment. *, *P*<0.001; †, *P*<0.001; ‡, *P*<0.001. Values are means ± SEM (n = 6).

The ERK1/2 pathway is a major downstream effector of TrkB signaling [27]. We examined the role of the ERK1/2 pathway in FTY720-P–mediated neuroprotection. Inhibition of ERK1/2 almost completely suppressed the neuroprotective effects of FTY720-P against oAβ–induced neurotoxicity (Figure 6A, oAβ+FTY720-P+1 µM U0126; Figure 6B and 6C, blue columns), whereas U0126 per se did not exhibit neurotoxicity nor enhance oAβ–mediated neurotoxicity (Figure 6C, green and gray columns). These results revealed that FTY720-P–mediated neuroprotection depended on ERK1/2 signaling.

## Discussion

This is the first report showing that FTY720-P is protective against oAβ1-42–induced neurotoxicity. We have demonstrated that FTY720-P enhances the production of BDNF, which activates TrkB and ERK1/2 signaling.

Although Aβ–stimulated mononuclear cells reportedly show increased mRNA expression levels of S1P_2_ and S1P_5_ [28], whether Aβ alters the expression profiles of neuronal S1PRs has not been reported. We demonstrated that neurons constitutively express all S1PR subtypes regardless of Aβ–stimulation. These results suggested that FTY720-P directly affects neurons via S1PRs. We had the technical limitation of evaluation for mouse S1PRs protein expression levels because well-characterized antibodies against mouse S1PRs are not commercially available at this time. Moreover, because we used primary cortical neurons in this study, additional experiments are needed to confirm whether S1PR expression profiles change in response to aging or various pathologic conditions.

BDNF and its receptor TrkB are widely expressed in the CNS [29]–[31]. These molecules play important neuroprotective roles, including contributions to cell survival, axon growth, neuronal transmission, and synaptic plasticity [32]–[34]. Several reports have documented that BDNF and TrkB contribute to long-term potentiation and memory formation [35]–[38]. Decreased BDNF-TrkB signaling induces deficiencies in spatial memory [39], whereas overexpression of full-length TrkB can enhance learning and memory [40]. Moreover, BDNF expression levels are lower in patients with AD [41], [42]. Thus, BDNF-TrkB signaling may play an important role in the pathology of AD. In this study, we demonstrated that BDNF-TrkB signaling is critical for the neuroprotective effects of FTY720-P against oAβ–induced neurotoxicity. We believe that treatment with FTY720 and resulting increases in BDNF expression are a promising therapeutic strategy for AD. Recently, FTY720-P was shown to improve spinal cord injuries via nonimmunologic mechanisms [43]. Furthermore, FTY720-P injections induced BDNF production and improved disease symptoms in mouse models of Rett syndrome [44]. These two papers corroborate the potential therapeutic utility of our approach.

In conclusion, FTY720 appears to be a promising therapeutic agent against not only multiple sclerosis but also various neurodegenerative diseases, including AD.
